# Understanding life satisfaction among the original inhabitants in the suburbanized areas at the outskirts of a major city: a qualitative study

**DOI:** 10.1080/17482631.2024.2350729

**Published:** 2024-05-09

**Authors:** Celestin Ndikumana

**Affiliations:** a Department of Governance and Public Administration, University of Rwanda, Butare, Rwanda; b School of Global Studies, University of Gothenburg, Gothenburg, Sweden

**Keywords:** Subjective wellbeing, life satisfaction, quality of life, suburbanization, urbanization

## Abstract

**Purpose:**

Suburbanization has become a major characteristic of urban development in sub-Saharan Africa, and shifting from agricultural-based areas modus vivendi to urban lifestyles affects subjective wellbeing of the original settlers. While there is lack of evidence in the literature of wellbeing in these areas, this study examines life satisfaction of these populations by means of individuals’ own appreciation and evaluation of quality of life.

**Methods:**

The study uses interpretionist and reflexive approaches, and analyses 76 interviews conducted through snowball sampling in two major suburbanized areas. Thematic analysis was used to analyse the data.

**Results:**

Generally, the findings show that respondents are satisfied with material living conditions due to improvement of availability of economic opportunities, roads and other transport services, social and community support. However, income inequality and urban poverty result in the inability to afford modern and high-quality urban living conditions, which creates feelings of vulnerability while limiting social relationships.

**Conclusions:**

There is a need to strenghten existing frameworks to fully respond to urban life requirements that relate to transport, education, hygiene, and sanitation services. It is also important to develop support systems that mitigate issues of gender discrimination, human rights, household decision-making, fashion, and cultural norms.

## Introduction and literature

Suburbanization continues to be a major characteristic of expansion of cities, but the concentration of populations in new urban areas does not always correlate with new jobs and availability of other necessary amenities for urban life (Buire, 2014; OECD/UN, & ECA/AfDB, 2022; Strauser et al., 2018). Instead, for the original populations, suburbanization corresponds with challenges caused by shifting from agricultural-based modus vivendi to urban lifestyles: unfavourable transport systems that may hinder the supply chain of transportation and logistics (Li & Wang, 2018; Melo & Graham, 2018; Shannon et al., 2017), socio-economic conflicts, degradation of livelihoods, forced expropriation and removal of poor residential units (Nduwayezu et al., 2021). To this, add the issues related to spatial segregation of populations (Xu & Zhang, 2017), lack of mutual social links (Sveda et al., 2016), as well as disruption of intracommunity relations (Biolek et al., 2017), all of which finally affect their quality of life.

In response to suburbanization and the overall growth of the cities on the African continent and elsewhere, a new approach has attracted the attention of researchers and policymakers: shifting the focus of any human development on the human beings with liberalization of personality at the core, instead of making the economic development as the centre of improvement of quality of life (Fabian, 2022; Wang et al., 2024). In this regard, one of the fundamental goals for most governments’ policies geared towards the welfare of citizens for the recent years has been life satisfaction (Kayonda, 2016; Miret et al., 2014; Stiglitz et al., 2009). This concept, generally referring to a cognitive and general appreciation of the one’s quality of life (Pavot & Diener, 2008), can be established either though independent indicators that do not consider individual evaluation, or through subjective indicators that capture the individuals’ own appreciation and evaluation of quality of life through existing social conditions around them (Nor-Hafizah, 2012). Normally, the latter approach relates to the broad concept of subjective well-being with the huge literature in emerging disciplines such as human ecology and public health (Das et al., 2020). Although there are different theoretical frameworks that guide empirical studies on subjective well-being (Diener et al., 1999; Fredrickson, 2004; Meadow et al., 1992; Michalos, 1980; Siedlecki et al., 2008; Wilson, 1960), this study is based on the evaluative theories that view subjective wellbeing as the way individuals compare their life conditions and circumstances with specific objectives or any subjective standards (Das et al., 2020). For instance, the social construction theory states that individuals may use peers as the standard for such comparisons, and higher subjective wellbeing will derive from the individual consideration of herself or himself as being better off than others. This can also be about how individuals make self-assessment of what should be the standard (Carp & Carp, 1982; Emmons & Diener, 1985; Michalos, 1980). Under this framework, the gap between how individuals perceive what life is and their thinking on how it should be, constitute the overall view of what subjective wellbeing is (Berger & Luckmann, 1966). In other circumstances, subjective well-being can be examined through the lens of adaptation range-frequency theory where the past of an individual serves as a means to set the standard (Brickman et al., 1978; Parducci, 1968). In that way, while life satisfaction is one of the major components of subjective wellbeing (Busseri & Sadava, 2011; Diener, 1984; Tov & Diener, 2013), it mainly refers to the extent to which an individual makes evaluations of the overall quality of the present life (Veenhoven, 2015). More explicitly, it becomes obvious that the primary focus of subjective well-being is the individual’s own judgement of how he/she perceives life and social conditions by self-reported data, rather than the reality constructed upon provision of official data by economic development indicators from governments and policymakers.

The links between urbanization and well-being have been examined in various studies to understand the changes in the lives of populations vis-a-vis their lifestyles and behaviour (United Nations Department of Economic and Social Affairs Population Division, 2019), and in relation with their life satisfaction (Seitsamo & Lmarinen, 1997). For instance, studies confirmed that positive life satisfaction is linked to urbanization by means of economic growth and development (Chen et al., 2019; Easterlin, 1970). Contrary to this, other studies came up with strong arguments that urbanization is negatively associated with life satisfaction (Graham, 2009; Winters & Li, 2017).

Although some studies have been undertaken to understand the quality of life in the suburbanized settings in developing countries (Alem & Köhlin, 2012; Duboz et al., 2021), available literature on this phenomenon in Rwanda relates to a few studies on life satisfaction of citizens in the country (Abbott & Wallace, 2012; Kayonda, 2016; Mousa Almatar et al., 2022). These add to reports on the scores of happiness in the country (Helliwell et al., 2016) or by establishing comparisons with other countries in the region (NISR, 2014; Veenhoven, 2015). However, none of the studies has linked the concept of subjective well-being with the rapid urbanization process that has been taking place over the last few decades. This results in lack of evidence for policy making and policy implementation in the context of suburbanization with regard to the populations who used to live in rural areas that later transformed into urban sprawls by means of suburbanization phenomenon. In this regard, the following questions remain at the centre of research on the process of suburbanization and subjective wellbeing in Rwanda: how do populations in newly suburbanized areas feel satisfied about general living conditions? How are populations under these areas satisfied according to their accounts of social needs and relationships with others? How are the populations in newly suburbanized areas satisfied with the potential for personal growth and relationships with externalities?

While lack of evidence of literature on subjective wellbeing is a critical issue along with huge growth of cities in sub-Saharan Africa in general and Rwanda in particular, it is important to note that Kigali City has undergone massive expansion in the last three decades. Indeed, this has been taking place along with other social and economic changes in the country due to structural reforms for economic growth that have been going on (World Bank, 2019). Such reforms are hinged on several strategies that include, for instance, the Economic Development and Poverty Reduction Strategies (EDPRS)-1 (2008–12) and the EDPRS-2 (2013–18), which stood as the country’s five-year flagship plans each aimed to sustain social and economic performance. While the country registered major achievements in terms of economic growth with an average of 7.2 under the EDPRS 1 and 2, these programmes were later transformed into the National Strategies for Transformation (NST) aiming to cement the requirements to meet the United Nations Sustainable Development Plans with the focus of bringing the country into the category of the Middle-Income Country by 2035 and the High-Income Countries by 2050 (Government of Rwanda, 2017). The recorded economic growth under EDPRS 1 and 2, coupled with the population growth, has affected the expansion of Kigali city with high registration of urban populations in the periphery of the city, with an average growth of over 28% per year (World Bank, 2019). Looking at the concentration of populations in the areas that used to be rural, this rate is by far greater than the annual urban growth of 4.5% per year (NISR, 2014).

While the expansion of Kigali has created growth of the city in terms of built‐up areas and infrastructure, it has at the same time made the populations who used to live in the rural areas in the neighbouring districts adapt their lifestyles to those of urbanites. As these populations navigate through new urban systems, they may face challenges related to transport, water, roads, and social integration. For instance, a study conducted by Gakuba (2020) reported that there were limited job opportunities, which pushes the populations towards new areas where they think they can make a living. This study seeks to understand how original inhabitants of newly suburbanized areas around a major city are satisfied with general living conditions, need for social needs and relationships with other people, personal growth, and spirituality. The study will expand the illumination on the links between suburbanization and subjective wellbeing in a developing country and will create insights for policymakers by constituting the basis for governance perspectives in terms of evidence-based decisions for populations who used to live in rural areas that transformed into suburbanized areas.

## Materials and methods

### Design and approach

The study adopts a case study research design with interpretations and reflexive approaches. The choice of case study design was based on the fact that suburbanization as an emerging phenomenon comes along with complex issues to the original settlers, hence the need to understand how different people in these locations are satisfied with such different situations in the real-life context. Semi-structured interviews were conducted between December 2022 and February 2023.

### Study setting

The study was conducted in two different urban sprawls around Kigali city: Masaka and Gahanga towards the East. Conceived in 1907 during the colonial period, Kigali became the capital of the country in 1962 due to its central location compared with Nyanza which was the seat of the king (Mwami) and Butare, which was the Belgian colonial seat. Figures by the National Institute of Statistics of Rwanda (2023) indicate that by the end of 2022 the population of Kigali was closer to 1,800,000, which is a very fast growth from a population of around 150,000 in 1991. The extension of the city since that period was characterized by general expansion in built-up areas and the compactness of the Central Business District (1990 to 2010). This was followed by the transformation of neighbouring rural areas into urban spawls, especially towards the eastern regions of the city due to the advantage of landscape which favoured the construction of residential houses, offices, schools, and other necessary buildings (REMA, 2013). As the city has been mainly expanding towards the East, the two locations of Masaka and Gahanga have been selected because they are under huge suburbanization and have been home of massive urban developments and lifestyles for a city of which the total area of 87% is urban. For instance, Masaka—and its surroundings—which used to be a rural area for more than 20 years back, was selected by the government to be the main country cargo terminal centre, expected to be the western end of Isaka (Tanzania)—Kigali Standard Gauge Railway (Magufuli, 2021). Similarly, Gahanga is a gateway linking the main city with several sites under suburbanization towards the new airport under construction, and which is expected to handle 14 million passengers per annum when all construction phases are completed by 2032.

### Population, sampling procedure and sample

The study was conducted among the original settlers of the two locations that have been under suburbanization for the last two decades. The concept of original settlers or original inhabitants is used in this study to refer to populations who used to live in these areas before the expansion of the city towards them. Some original inhabitants being the owners of land—which constitutes the major income for their farming and agricultural activities but also needed by newcomers and sometimes at high price—consider selling it as a new opportunity for financial resources. Before Masaka and Gahanga were transformed into urban sprawls around Kigali city, their populations lived in a rural life setting until 2004 when the city of Kigali started expanding over different directions.

The study considered respondents from the households that were settled in these locations by 2005. The study being qualitative in nature, respondents were reached through snowball recruitment. For the matter of conformity, one individual was interviewed in the household, and only the person identified as a head in the household could be reached out for interviews. The information provided, however, was not considered as representative of the collective feeling of the household, but as a personal submission of life satisfaction on different issues regarding the suburbanization phenomenon. In total, the study analysed the data collected from 76 interviews, and the maximum number of respondents to participate in the interview was determined through the principle of data saturation.

### Instruments

Interview guides were developed for data collection. The interview guides inquired about satisfaction/dissatisfaction with material living conditions, happiness/unhappiness, social needs and relationships, potential for personal growth and externalities, and spiritual and religious activities. The demographic characteristics were captured for each respondent before recording the interviews.

### Data collection

Before data collection started, the researcher and research assistant first contacted the local administration leaders who were at the same time involved in identification of respondents based on the inclusion-exclusion criteria. Once potential participants accepted to take part in the study, they were recruited and requested to sign consent before interviews. Interviews were conducted in the local language and lasted between 25 and 30 min each. Interviews took place at the study participants’ homes, and the interviews were digitally recorded. The audio recordings were removed from the recorder and were saved in the researcher’s computer, and they were later transcribed into textual data for analysis.

### Trustworthiness of the data

To ensure trustworthiness of data and to adhere to the principles of credibility, authenticity, transferability, dependability, and confirmability, the researchers undertook important steps applied for such a purpose in qualitative research (Guba, 1981; Moradi et al., 2020; Shenton, 2004). The researcher developed the instrument basing on existing literature. Counter verification of the data collection tools was done to ensure that all elements were captured. During the data collection process, the researcher referred to probing mechanism in which all possible details on the data could be explored. The research assistant ensured paraphrasing of each respondent’s submission during the interview session to maximize assurance of data correctness. In addition, all details that can permit comparisons of the study results with other studies with similar findings and the understanding of the study findings were provided: participants and sampling methods, design and approach, and the procedure for data collection and analysis. Moreover, to remove some inconsistencies that would be caused by respondents not being aware of the main changes that have been taking place in the area under the study, the main inclusion criteria for participating in the study was a settled inhabitant of the study locations by 2005 which marks a major step in the massive expansion of the city towards its outskirts. The researcher enhanced reliability by checking for the stability of responses throughout the interview datasets, and this was enhanced by the use of field notes along with recording the interviews into digital files that were later transcribed into the word texts.

### Data management and analysis

To get a sense of the main issues contained in the data, all recorded transcripts were subjected to intensive readings. Transcribed data were analysed by use of mixture of inductive and deductive coding mechanism that aimed to consider the main elements to be focused on under the indicators of life satisfaction. This was done to identify deductive thematic realizations under the study. Specific themes were explored with inductive coding to make comprehensive nuances, and cross-checks over these nuances was accomplished for thorough verification and validation purposes. As there were different categories under which themes were grouped, the overarching themes under the elements of life satisfaction were put together based on their similarities and differences. To develop a more comprehensive understanding of the themes under the phenomenon of life satisfaction in the context of suburbanization, triangulation of the data was referred to through peer debriefing and reflexibility.

### Ethics

Prior to undertaking data collection, a research clearance was provided by the Institutional Review Board. Later the authorization to interact with the study participants was requested from respective local leaders upon presentation of the research clearance. The study was based on the principle that at least one person from a household involved in the study could participate in the study. To ensure the study participants understand the purpose of the study, objectives and procedures were explained to them and they were requested to sign an informed consent before participating in the interview. Participation in research was voluntary and participants could withdraw from the study at any stage they wished to do so. The research ensured confidentiality and privacy of respondents by use of codes for any reference purposes, and the process of data collection involved local leaders to allow identification of respondents. All ethical measures were shared with research participants prior to signing the informed consent.

### Limitations

The study presents some methodological limitations as, for instance, it could not be possible to predict the sample size before, or base on the random sampling methods. The fact that we based the sample determination on snowball sampling methods could lead to weaknesses that a chain of respondents with the same characteristics would fall among the study participants for interviews. To overcome this challenge, it was ensured that the questions were asked with a maximum credit of carefulness so that no single information could remain unnoticed. Similarly, it is possible that some respondents could not disclose the real information on some issues, and this was mitigated by creating trust between the researcher and the study participants before the interview activity could start.

## Results

### Demographic characteristics of respondents

There were more male than female respondents. According to the recent housing and population census (National Institute of Statistics of Rwanda, 2023), female heads of households in the country are around 30%, although this category of population constitutes closer to 52% of the total population. Most study participants were aged between 46 and 60, 90% of them being in the category of married people. With a population of 65% under the age of 30% by the end of 2022 (National Institute of Statistics of Rwanda, 2023), the study results present presumption that younger couples from the study sites prefer to live in other areas that might be more affordable for them. While closer to 20% of respondents reported to have no level of education, nearly 60% among them attended primary school. The situation is better than the average figure on education uptake in the country considering the recent statistics indicating that 22.3% and 54% of the population have either no level of education or a primary education, respectively. It is assumed that the level of education in the areas under suburbanization is slightly higher compared to other remote rural areas, as the former are located closer to the major city with more education, infrastructure and services. Results show a slightly smaller number of individuals undertaking agricultural activities in suburbanized areas, compared with the national average of 40% of people who are involved in the sector of agriculture. Although a third of individuals who participated in the study practice their own business as a major occupation, there is an indication that a considerable number of people living in agriculture are still high in the context of an urban setting. Table I indicates demographic characteristics of respondents.

**Table I.** Characteristics of respondents (*n* = 76).

Variable	Frequency	Percentage
Gender		
Male	63	82.90
Female	13	17.10
Age category		
Below 40	4	5.3
Between 41–45	7	9.2
Between 46–50	13	17.1
Between 51–55	19	25.0
Between 56–60	23	30.3
Between 61–65	6	7.9
66 and above	4	5.3
Marital status		
Single	2	2.6
Married	69	90.8
Separated	2	2.6
Widowed	3	3.9
Education status		
None	13	17.1
Below primary	23	30.3
Primary education	21	27.6
Post-primary	5	6.6
Secondary education	11	14.5
Post-secondary	1	1.3
University	2	2.6
Occupation status		
No occupation/unemployed	4	5.3
Farming	28	36.8
Business (own)	23	30.3
Civil servant	8	10.5
Private sector	11	14.5
Retiree	2	2.6
Size of household		
2 and below	21	27.6
Between 2–5	46	60.5
6 and above	9	11.8
Type of accommodation		
Own house	63	82.9
Rented	10	13.2
Social or Borrowed	3	3.9

## General life satisfaction

The study focused on subjective views, accounts of feelings and experiences of satisfaction or dissatisfaction with living conditions of inhabitants who used to live in the study locations before transforming into urban sprawls around a major city. The real-life stories from respondents were analysed under the concepts that relate life satisfaction.

### General living conditions and health, hygiene, and sanitation

Stories from the study participants indicate that they generally feel satisfied with material living conditions because of socio-economic changes that occurred in their lives since the areas became urban. Respondents reported that satisfaction with living in an urban area is a result of being closer to water and electricity infrastructure and owning a good house. In the context of economic transformation and, in comparison with rural setting, life satisfaction with living in an urban setting is associated with the improvement of personal living standards in terms of access to the infrastructure and services, availability of different economic opportunities that include undertaking commercial businesses, social interactions and employment. One respondent mentioned that:

… yes, I am happy with it, although I am getting older. We used to live in simple housing structures but now we have put up modest houses that align with urban life. You see when urbanization progresses, people benefit from that. Look … like my children have been able to get jobs and they have built their own houses and can support their families because of that job. I am an old woman and don’t work but can live because of my children’s jobs Like now my neighbors are well-off and when one needs to take a patient to hospital, you may call them for a ride to hospital. That’s the same when there has been an incident in the family, like death, they may support financially for a burial.

The population under current urban settings also reveal that suburbanization came with modernization and a shift from existing practices in the living conditions. This can be considered as a change in the mindset with adoption of urban lifestyle, as it was reiterated by the respondent who narrated that:

… life was not that easy at the time. Even those who had money did not care about some good practices like buying shoes. I can remember when we had church-related ceremonial events, and we could go barefoot. That was bad, and it was a rural life thinking. But I don’t know if there is child who can accept to go to such a ceremony or even to school without shoes on. That means in the current setting there is a clear vision, there are some very good things that take place in the changes of our mindsets …

Life satisfaction with living conditions was also linked to living in a clean environment, the improvement of hygiene and sanitation due availability of water. It was reported that “as you can see, living in a good and clean environment and having access to water gives you a guarantee of getting away from hygiene-related diseases that generally attack people in rural areas”. In relation with sanitation and health, again, living in a condensed settlement has boosted the awareness for individuals to stick to their contributions for community health insurance schemes, which provide access to easy and rapid healthcare service delivery.

… we have a healthcare and a hospital in this area. You see it is a big and good hospital. Most people here are under a community health insurance scheme which gives them access to healthcare services. It gets easy for the leaders to mobilise the population on their contributions because they live in an urban setting … communication is not difficult.

The transformation of the rural areas into the urban areas is reported to have come with some other challenges among the original inhabitants. Under the urban living lifestyle, some individuals perceive life hardship in relying on the agriculture as the source of living yet amidst of scarcity of land which used to be an important source of wealth. This seems to lead to the challenge of food security unlike when “we used to be food secure with different types of food commodities—you are also aware—today we do not have enough land for agricultural activities and therefore resort to buying our own food commodities … and yet we don’t have jobs. That’s a problem … it is a challenge which is associated with this urban life that is expanding in these locations”. In the context of urban setting, the situation translates into vulnerability of individuals whose job opportunities are very limited and remain with no other option than relying on agriculture which is practiced on plots of land.

In other circumstances, those who are dissatisfied with the current housing and the status of hygiene mention lack of financial means to be able to live in clean places, but still with the enthusiasm to improve the current situation. One respondent reported that “… yes … water is expensive, and I can’t afford using it as I would wish. But I am very anxious about this. And, you see, my neighbour here has a very nice and modern house. I would wish to live in a nice house like this one, but I can’t because I have no financial means”. Another one reiterated the “… my financial capacity is limited to this house. So I can say that I am happy with what I have now, but any time I get an opportunity, my wish is to live in a nicer house and a more clean environment”.

### Governance: policy decisions, conflict and security

The study investigated respondents’ satisfaction with governance practices in the newly suburbanized areas. All in all, it emerged from respondents that the transformation of the areas into urban places does not degrade the original residents’ land property rights as the selling of land is concluded between the landowner and the buyer and is facilitated through existing land transfer mechanisms, and in line with related laws in place.

The current suburbanization process takes place in accordance with the city master plan with the type of construction facility in each area. In most cases, the local administration initiates and facilitates the site planning before the deals for selling and buying plots can start, which may increase the value of land: “… there is a site planning going on over there. This is being facilitated by the administration according to the city master plan. The selling of plots takes place later once the roads have been marked. Before the master plan was developed, there could be conflicts created by lack of agreement between the landowners in case of public infrastructure like a road”. One of the respondents said that this was not the case before: “… my neighbour wanted his plots to have more value and created a road that crossed my land. This resulted in having my plot reduced, and it has been a big loss for me”.

While some individuals are aware of the usefulness of existing policies regarding land tax because of their contribution to the economic development, some people stay unaware that they owe land taxes to government until when they are required to conclude land transfer after selling the plots. To this end, it was learnt that “I am aware of such policies like land tax, but I think I cannot complain about them because such taxes contribute to economic development by providing us with basic infrastructure like hospitals, roads, markets, etc …”. But another respondent said that “… nobody has asked me about that, but I know that this will happen. It happened to my neighbour who was requested to pay a big amount of taxes at the time of transfer, which they did not know before”.

The transformation of rural areas into the urban sprawls is linked with improving security attainment among the inhabitants because of easier strategic position for security measures and rapid intervention in case of security threats. The local administration having security as a primary focus, there is less difficulty to oversee security matters in the urban areas than rural settings. One of the respondents informed us that:

The fact that this area is transforming into an urban site has brought so many advantages to us. The first thing I can tell you is security. Leaders of the local administration have no difficulty with tightening security measures because we live in grouped settlements. We feel comfortable about security in the current set up.

The implementation of some policies for the requirements of urban settings, however, in relation with hygiene creates challenges to some residents with low financial means. Consequently, for sustainability of policies related to garbage collection, some individuals are pushed to supporting the burden by paying higher fees for the service because of a considerable number of people who cannot afford the related cost.

… so they come and explain. And we are requested to register with a particular company that deals with garbage collection here. Once some people are unable to pay for services, they withdraw from the arrangement. If the company had set a certain amount for service per household and they realise that a big number is no longer contributing, they resort to setting a higher amount for compensation to cover the cost of services. Then it becomes hard for some who can pay for the service.

## Income and other sources of living

Most respondents recall that the main source of income in the rural-life setting came from land in terms of agricultural products and livestock. Although not modernized, agriculture and farming were major entries for jobs even for those who do not own land in a form of land leasing or as being employed in the agricultural sector: “I did not own land, but I could lease some and could plant some agricultural products that I lived on as food or got some money from selling them”.

While some individuals still rely on agriculture as a source of income in the urban setting, it is not so beneficial for them because it is done on a small-scale area as most land has been sold out, which does not permit sustainability in food security:

“I used to live on agriculture and farming, but at the time there was abundant land. It is now impossible because we don’t have much land for agriculture. We just do it on a small scale and it doesn’t give us many benefits. The land is being used for residential purposes and agriculture has very little value now.

In comparison with rural life, however, most respondents reported being happy with the availability of different opportunities that serve as sources of income in the rural setting, but with challenges resulting from the fact that life gets more expensive, and that money depreciates all the time: “ … of course the income has increased as we can have more opportunities. Some of us have engaged in conducting businesses and other activities, but the cost of living keeps increasing all the time, the value of money is no longer the same”.

For transport and communication in the urban setting, the general view from the study participants is that suburbanization has facilitated access to transport facilities due to the expanded development of the road infrastructure. Public transport being more affordable, it stands out to be realized that there are major transport companies which operate the routes that connect the study sites to the city and the rates, fixed by the regulator, are considered fair. One respondent reiterated that “we get a bus from the main station. It can take you to town and other areas. There are different buses. Or else if you want to go fast, you can try a motorbike, but it is more expensive … it depends”.

In line with satisfaction with education services, a general view from the study participants’ stories translates into dominance of satisfaction with availability of education infrastructure for basic education purposes. In fact, it was indicated that the distance between residence and schools has reduced considerably for the last few years: “children are lucky because the government has built many schools. There are public and private schools around here, and the cost of education does not cause any threat for learners in primary schools”. Although respondents argued that public schools were affordable, private ones were described as expensive and with better quality, yet very few among respondents had confidence that they could send children to them. Therefore, income inequalities across different levels of populations create life dissatisfaction with the choice of schools for children’s education for some populations. One of them mentioned that:

You have to choose the school depending on your financial situation. You see … there is private school run by the nuns over there, and children who attend to it are fluent in foreign languages. Private schools are good. My daughter cannot speak those languages like them. But I take my children to public schools because I cannot afford the expensive ones.

## Interactions, relationships, personal growth, and spirituality

At community level, the issue of relationships is appreciated by many study participants who observe a strong bulk of relationships that has been created by households living closer to one another in the urban settlements. This relationship, which is often strengthened by community support in case of a social event or a social need, creates a feeling of a sense of belonging to the urban community. It is argued that this has contributed to social interactions through different events like *Umuganda* (Community works organized at the end of every month), and social events. The observation of one interviewee was that social events have been facilitated by easy communication in the urban setting: “Everybody feels pleased with that. Communication is easier in town than in the rural area. People socialize a lot when there is an event like wedding or when we participate in community work. Another one reiterated that:

Social interactions have increased since this area has transformed into urban area. The relationships have become improved because even though you live closer to one another, but everybody cares about their own business. But in case you need them or if there is a social event and you invite them, they will join you. If they also have something that needs a social gathering, they will let you know. We can also meet at the end of the month during the *Umuganda*”.

The urban lifestyle, on the other hand, has come with other practices that were not part of the usual life in the rural setting. Such include, for instance, more planning and careful selection of participants to social events due to constraints of budget in the urban setting.

“to have a big number people participating in social events, that was because rural life was easier than today’s. If you have to plan for a social event, you need to observe what current life dictates you … like thinking about the people you need to invite depending on the budget you have. You need to work out expenses that will be incurred. That did not happen before because we had everything from our farms, but now you need to buy everything”.

The creation of urban sprawls from the areas that used to be rural, also, resulted in having new populations inhabit the new urban sites, closer to the original inhabitants, who had lived there for a long time, and in most cases the owners of land. While most old inhabitants feel that the relationships with new ones are easier to create, some few respondents had the feeling that the newcomers are supposed to be the icebreakers of such social interactions: “it can be all right going to their home, they will welcome me. But I think that they should introduce themselves to me first … you know there is a feeling that I am not as rich as my neighbour, so it doesn’t sound good of me keep going to their home”. This gives the impression that the economic inequality between existing and new inhabitants may be a barrier to engagement in social relationships.

At family level, the shifting from a rural to an urban lifestyle is appreciated by respondents because of new opportunities as some of them can find jobs and venture in commercial businesses. However, it was revealed that some conflicts emerge among the spouses because of disagreements on land utilization.

Let me give you an example. There might be a conflict caused by a disagreement in utilization of land. One spouse might need to sell it and relocate to another area in disagreement with the other. So, that can be a problem. It becomes worse when the money is finished up and the family doesn’t have a place to stay. We have some who sold their lands and left but came back later. They live with conflicts.

In addition, living in the urban setting was reported to have resulted in changing of partner’s behaviour. It was mentioned that “ … it is not all roses everywhere in some families they struggle because the wife has gotten so much money and starts disrespecting the husband and vice versa”. In terms of human development and expansion, this can be interpreted as having women becoming more empowered and independent as result of being exposed to urban lifestyles. This is mainly due to the fact that living in traditional rural areas is associated with gender issues where men exercise more powers upon women. Shifting to the urban life may therefore, as it appears in the submissions of some respondents, lead to the reduction of the risk of gender violence. The creation of disagreements between the two social groups culminates in the dissolution of marriage for some couples which fail to embrace the issue of gender dynamism as it came out from a male respondent who said: “We have such problems. There are so many disagreements and this time we are confronted with no other choices than seeking divorce. The urban life is pushing families to such challenges that we were not used to”.

The expansion of city towards the areas that were used to be rural also has been a considered, by some individuals, as a source of deviation from cultural norms, but which to some others is considered as moving with modernism and fashion. To this point, while some study participants feel unhappy with the types of clothing among both the adults and the youth, others view it as a lifestyle that changes with time. It can be noted, however, that most study participants feel uncomfortable with high rates of pregnancy and other unpleasing behaviour that they feel is a result of urban lifestyle. One respondent put it that “for us we don’t feel good with young people who get away from our culture and adopt a foreign culture”. In line with what seems to be an expansion of human development—but considered by individuals as deviation to culture—another one added that

On that topic we need to strengthen our culture. Because these days you see grown up women dressed in miniskirts, young men with dreadlocks smoking weed and men getting tempted and acting out of character and that is no way close to our culture. We need mobilization on this specific topic because young people even adults are adapting behaviors that don’t align with our culture.

This reflects the multidimensional nature of urbanization which marks a social migration into a different social setting. In fact, the consideration of individuals’ history, their surroundings, practices, and all living lifestyles being the constituents of their culture, it may become hard with some people who lived for a long time and who are exposed to some urban practices. Urban lifestyles can therefore not only affect the communities’ demographic composition of the areas but also have some influence changes in on their cultural fabric.

In relation with religion and spiritual practices, respondents affiliated to religious communities reported to be happy with their affiliations, but with some discomfort caused by the spread of so many churches around the community. The degree of dissatisfaction with such churches a consideration is that some of them are associated with scamming: “we feel very uncomfortable with this. I was born in a family affiliated to the religious community. But telling me that joining a religious community will make me rich is not believable. Most of time you are scammed by telling you that you will get richer”. In that view, some new religious communities were considered be a source of conflict among family members: “I was telling you that we see it coming and we don’t feel well about it. Can you imagine you have a property at home and your partner belonging to the church takes it to them in a form of offerings or your spouse takes the money to them thinking that they acquire blessings in return! It is a scam”.

## Discussion

This study has evaluated the status of life satisfaction among the original populations in the areas under suburbanization around a major city of a developing country in sub-Saharan Africa. It is based on the argument that economic opportunities that come along with living in urban settings should be the foundations of social development and human expansion that liberalize individuals from poverty, inequality, exclusion, and vulnerability. While evidence shows that studies about subjective wellbeing are very rare in developing countries (Duboz et al., 2021), there is a huge literature on variation in good life between rural and urban areas in North America and Europe (Helliwell et al., 2016). In these countries, urban life creates lower levels of satisfaction than rural life (Berry & Okulicz-Kozaryn, 2011; Sørensen, 2014), which becomes the opposite in developing countries -as for the case of this study- where economic opportunities and social support are associated with urbanity (Easterlin et al., 2011). In fact, living in a rural area is characterized by hard life due to lack of basic amenities like roads, water and electricity, and services. Therefore, studies of well-being in developing countries show very low happiness scores associated with living a rural life as people’s goal attributes more value to urban life (Helliwell et al., 2016).

In fact, unlike in developed countries where people in rural areas live on other means than farming (Millward & Spinney, 2013) while at the same time enjoying the quiet environment with less negative externalities, satisfaction with suburban life in sub-Saharan Africa in general—and in this study, particularly—is explained by changes in economic conditions in terms of economic opportunities and access to infrastructure and access to different services (Duboz et al., 2021). The economic opportunities in everyday life are expected to be better through self-reported assessment of life satisfaction on the different elements of material living conditions and social relations, like in other studies that have examined the same topic (Macia et al., 2010).

While suburbanization process in developed countries mostly involves the movement of people from big city centres because of congestions and other externalities, suburbanization in developing countries is highly influenced by rural–urban migration as majority of populations in these countries live in rural areas. All in all, suburbanized areas remain under an urban lifestyle in both developed and developing countries. The findings of this study have confirmed that in some circumstances, suburban life in developing countries does not only increase population levels of happiness in terms of material conditions but also attract domestic comfort, and other resources that are of administrative, political, informational and organizational concern (Pandas, 2020).

In the current study, this comfort relates to changes in the living conditions due to availability of infrastructure and services that would make life hard in rural areas. Living in suburbanized areas is appreciated for the improvement of hygiene and sanitation due to a high coverage of penetration of water infrastructure in the city and its direct outskirts. Although the use of improved sanitation facilities is currently estimated to be at a percentage of more than 85% among urban and rural areas, available figures show that a big share of populations having access to such facilities are in urban settings (Twagirayezu et al., 2023). The findings of this study show, however, that some populations may be exposed to poor hygiene and sanitation practices due to financial constraints. The country commitment to scale up enhanced sanitation and water to 100% of all households by 2024 (Ministry of Infrastructure, 2018b) should go along with the efforts to maximize the affordability of water and sanitations services.

The availability of electricity in urban areas compared with the rural areas makes populations living in the study settings feel more comfortable and more satisfied, because of the advantages that come along with the development of electricity infrastructure and its availability. Such an observation can be linked to the efforts of country to connect households to electricity to as many households as possible for the last two decades. The World Bank report (World Bank, 2023) shows that more than 90% of people living in urban areas in Rwanda have access to electricity. Satisfaction with health services may be a result of a number of policies that have been undertaken in the health sector for the last couple of decades, including the community health insurance scheme which has tremendeously revolutionalised access to health services in the country (Collins et al., 2016).

Shifting to urban lifestyles has also permitted the populations to live in more grouped settlements for enhanced security due to rapid intervention towards any security threat. This comes along with other advantages of decentralization strategies by which security is ensured at the grassroot level. In fact, the administrative structure of the country is strongly tied with security governance. According to the results of this study, such a structure is considered as a source of strong security patterns in the urban area. A study conducted by Barihuta a few years back (2017) reported that Kigali and its surroundings enjoy the security of persons and goods as a result of security mechanisms at the very low levels of administration due to a community security-led mechanism locally known as the *Irondo*.

The study findings also highlight the satisfaction with the availability of education services, which reflects one of the major achievements in the education sector for the last few years since the introduction of 12-year basic education in 2012 (Endres et al., 2023). Such a policy has been supported with a heavy investment in the development of infrastructure including building of new schools near local communities and other necessary amenities for the education services. The fact that some study participants reluctantly express their satisfaction with having their children join public schools translates into the need for these schools to shift the focus on the quality of education acquired from them.

The issue around governance of land is at the centre of life satisfaction with regard to individuals who used to live in these places under a rural structure. While land is a major property for these populations, it is obvious that its ownership decreases over time as much of it is used for residential and public buildings, and other major infrastructure to fulfil the requirements of suburban setting. The constitution of the country provides the non-encroachment of the right to property including land, except where it has to be used for public interest—and in accordance with existing laws (Norwegian People’s Aid & Rwanda Civil Society Platform, 2017). While an agreement for selling and buying land is concluded between the buyer and the seller, the laws define the circumstances under which the expropriation is conducted for the public infrastructure. The fact that some dissatisfaction regarding land governance was found out concurs with intra-family disagreements and disputes on land use and transfer, as well as shortcomings in the land transfer process. The present findings also concur with other previous findings on sources of conflicts regarding land use (Legal Aid Forum, 2015).

In terms of transport and communication, it is obvious that availability of road and transport infrastructure in general creates more vibes of happiness among the study participants. Relative to what used to be the situation when the areas were rural, the study findings fall in line with developments in the improvement of the transport sector in the country for the last few years. Recent investments in the building of new roads, maintenance of existing road network and attempts to improve efficiency in the transport system have been the focus for the government despite the issues road traffic management, delays and unpredictability in transport facility, and integrated public management systems (Ministry of infrastructure, 2018a). The satisfaction of study participants with existing channels of communication is attributed to the recent developments in the communication sector in the country, which in 2023 had reached the level of at least one member having mobile phone among 80 percent of households in the urban area (National Institute of Statistics of Rwanda, 2023).

While living in urban areas has brought the populations to live in compact settlements, there is a reluctance among the study participants to believe in the atmosphere of social relationships in urban life at the equal feet as in rural lifestyles. In fact, while some practices like the community development work (umuganda) are embedded in the Rwandan social policy perspectives, other social gatherings like wedding have a different design for both urban and rural settings because of financial implications that may be attributed to them in the urban areas.

Despite the status of life satisfaction with areas having transformed into urban settings due to economic opportunities, it is important to consider that much caution is needed by policymakers in the country to have control on the acceleration of challenges that have always evolved with suburbanization in developing countries, especially in sub-Saharan Africa (United Nations, 2020). Some of such concerns in this study include, for instance, good urban planning and management which could result in the improvement of the quality of life due to better education, health, and sanitation services. A focus on better urban planning could also mitigate the issue of land administration and management in order to avoid homeless populations once all their land has been sold.

In relation with the study findings, again, satisfaction with the quality of life is much more concerned with inequality of income in the urban setting. The study results constitute yet another evidence that the links between suburbanization and income inequality in sub-Saharan Africa have remained persistent over a long period of time. This has attracted the attention of scholars to call for governments to craft mitigation strategies that respond to the financial needs of populations who migrate to the urban areas (Sulemana, 2019). Addressing income inequality would contribute to the equitable share of available resources by allowing more households be able to pay electricity, transport and service fees (garbage collection). While income inequality and urban poverty results in inability to afford living conditions and inability to live in the modern quality urban requirements, this may also create feelings of vulnerability among people with less financial means, which affects social relationships and interactions with other populations.

The differences in gender dynamics between rural and urban areas are reflected in the findings of this study. It was observed that shifting from rural to urban lifestyles has exposed some social groups to expand their mindsets on how they view the world when it comes to gender and culture. This seems to have created a shift in the decision-making and ownership of property, as well as the cultural perspectives. To create frameworks that cover the integration of populations towards the urban lifestyles, there is a need to focus on support systems that mitigate issues of gender discrimination, human rights, family conflicts (household decision-making) and by which populations can be able to manage changes in fashion, cultural norms and behaviour in the urban lifestyle.

## Conclusion

The study sought to understand life satisfaction of populations under suburbanizing areas around Kigali city in Rwanda. The study findings show that shifting to urban life has created more satisfaction among the study participants as they were comfortable with material living conditions in terms of availability of economic opportunities, availability of roads and transport facilities and services, social and community support. Less satisfaction among the study participants was linked to income inequality and urban poverty, which create feelings of vulnerability while limiting social relationships. The study suggests that there is a need to strengthen existing frameworks to fully respond to urban life requirements of transport, education, hygiene, and sanitation services. In addition, there is a need for development of support systems for mitigation of gender discrimination and human rights issues, and which respond to family conflicts and cultural norms that evolve along with shifting from rural-life modus vivendi to urban lifestyles.
